# Online Extraction–DPPH–HPLC–DAD–QTOF-MS System for Efficient Screening and Identification of Antioxidants from *Citrus aurantium* L. var. *amara* (Rutaceae): Integrating Sample Preparation and Antioxidants Profiling

**DOI:** 10.3390/antiox11051014

**Published:** 2022-05-20

**Authors:** Yecheng Xiao, Fuhua Fu, Youhe Wei, Shuyun Shi, Yang Shan

**Affiliations:** 1 Longping Branch Graduate School, Hunan University, Changsha 410125, China; xiaoyecheng321@163.com; 2 Hunan Agriculture Product Processing Institute, Hunan Academy of Agricultural Sciences, Changsha 410125, China; fhfu686@163.com; 3 Natural Product Research Laboratory, Lianyuan Kanglu Biotech Co., Ltd., Lianyuan 417100, China; weiyouhe123@163.com; 4 College of Chemistry and Chemical Engineering, Central South University, Changsha 410083, China

**Keywords:** OLE–DPPH–HPLC, QTOF-MS, *Citrus aurantium* L. var. *amara*, antioxidant, flavanone

## Abstract

The lack of a direct connection between solid edible or medical natural products and bioactive compound profiling is a bottleneck in natural product research and quality control. Here, a novel integrated system, online extraction (OLE)–2,2′-diphenyl-1-picrylhydrazyl (DPPH)–HPLC−DAD−QTOF-MS, was fabricated to extract, screen, and identify antioxidants from the whole fruit of *Citrus aurantium* L. var. *amara* (CAVA, Rutaceae) simply, rapidly, and efficiently. The system consumes less sample (1.0 mg of CAVA powder) and requires a shorter analytical time (45 min for sample extraction, antioxidants screening, separation, and identification). Eight antioxidant flavonoids were screened and identified, and six available flavanones were sensitively, precisely, and accurately quantified. Two major flavanone glycosides, naringin (50.37 ± 0.43 mg/g) and neohesperidin (38.20 ± 0.27 mg/g), exhibit potent DPPH scavenging activities with IC_50_ values of 111.9 ± 10.06 and 178.55 ± 11.28 μg/mL. A minor flavanone aglycone, hesperitin (0.73 ± 0.06 mg/g), presents stronger DPPH scavenging activity (IC_50_, 39.07 ± 2.51 μg/mL). Furthermore, density functional theory calculations demonstrated their electron transport ability and chemical reactivity, which confirmed the screened results. The results indicate that the developed OLE–DPPH–HPLC−DAD−QTOF-MS system provides new perspectives for analysis of antioxidants from complex natural products, which also contribute to the quality evaluation of CAVA.

## 1. Introduction

Free radicals are usually produced in normal human metabolism, and excess free radicals can be toxic and cause some chronic diseases, such as diabetes, Alzheimer’s disease, cardiovascular disease, and even cancers [1,2]. Antioxidants can scavenge free radicals, and to some extent, postpone or avoid the onset of free-radical-related diseases [3]. Natural products (e.g., fruits, vegetables, plants, herbs) have been considered as significant and vital resources for antioxidants [4,5]. Hence, the investigation of antioxidants in natural products has become a major topic in the field of natural product research [6,7,8]. Traditional antioxidant activity-guided isolation and evaluation procedures have yielded valuable findings; however, the complexity of natural products makes the work labor, cost, and time-consuming, and some antioxidants might be lost because of decomposition or dilution effects [9]. Therefore, it is necessary to fabricate a simple, rapid, and efficient system to systematically screen and identify natural antioxidants.

Ferric reducing antioxidant power (FRAP) assay, 2,2′-diphenyl-1-picrylhydrazyl (DPPH) and 2,2′-azino-bis(3-ethylbenzothiazoline-6-sulphonic acid) (ABTS) radical scavenging abilities have been selected to evaluate the antioxidant activity of a single compound or crude extract [9]. Luckily, in the last decade, high-performance liquid chromatography (HPLC)-based separation technology online coupling with post-column FRAP/DPPH/ABTS assays have been developed and successfully applied to rapidly and robustly screen antioxidants from natural products [10,11,12]. In the online hyphenated methods, FRAP reagent, DPPH, or ABTS was pumped into HPLC post-column flow, and peaks with antioxidant activity can be detected by reducing absorbance, which made evaluating the antioxidant activity contribution of each HPLC peak to crude extract possible. However, variable HPLC mobile phase compositions influenced the post-column reaction, lower sensitivity and resolution will present for peaks with less content or weak antioxidant activity, and the continuous post-column flow of FRAP/DPPH/ABTS will consume more reagents [13]. Comparatively, offline mixing DPPH with crude extracts coupling with HPLC provided a smoother baseline, higher sensitivity, and simpler operation [14]. Nevertheless, the mentioned methods still consumed some labor, time, energy, and reagents to prepare crude extract solutions (e.g., heating reflux/ultrasonic/microwave extraction, concentration, dissolution) from the relatively large amounts of samples. Notably, our groups successfully developed guard column-based online extraction (OLE)–HPLC technology, and in the OLE system, only a guard column packed with 1–2 mg of solid sample was needed, and a single injection realized the sample extraction and analysis of complex natural products [9,15]. Thus, integrating OLE–HPLC online with DPPH mixing to fabricate an OLE–DPPH–HPLC system would have overwhelming advantages, by which the extraction, antioxidants screening, assessment, and analysis would be simultaneously obtained.

Peels, juice, and seeds of the *Citurs* genus present excellent antioxidant activity because of the existence of phenolic compounds [6,7,8]. *Citrus aurantium* L. var. *amara* (CAVA) is a variant of *C. aurantium* L., which has been consumed as an edible and medical resource for regulating Qi, strengthening the spleen, relieving asthma, and reducing sputum [16]. Flavonoids are the main bioactive compounds in CAVA [17], and pharmacological research of crude extract, the bioactive fraction of single compounds indicated the antioxidant, anti-inflammatory, antiobesity, antivirus, and antitumor activities [18,19,20]. To date, antioxidant profiling analysis of CAVA is very limited. Thus, the development of a facile, rapid, and efficient method to comprehensively investigate antioxidants in CAVA is in high demand to clarify their therapeutic effects. Therefore, this study established the OLE–DPPH–HPLC−diode array detector−quadrupole time-of-flight mass spectrometry (OLE–DPPH–HPLC−DAD−QTOF-MS) system to investigate the antioxidants in CAVA. After that, eight antioxidant flavonoids were screened and identified, and six commercial flavonoids were selected for validation and quantification. Later, density functional theory (DFT) calculations were fabricated to evaluate the electron transport ability of antioxidants, which further validated the screening methods. The results indicated that the online system was rapid, reliable, sensitive, accurate, and applicable for antioxidant evaluation of complex natural products.

## 2. Materials and Methods

### 2.1. Materials and Reagents

Chromatographic reagents (i.e., methanol, formic acid) were purchased from Sinopharm Chemical Reagent Co., Ltd. (Shanghai, China). DPPH was bought from Sigma-Aldrich (Steinheim, Germany), and the freshly prepared DPPH solution was kept in the dark. Six standards, narirutin, naringin, hesperidin, neohesperidin, didymin, and hesperitin with purities over 98% were obtained from the National Institutes for Food and Drug Control (Beijing, China). Other analytical chemicals were from Sinopharm Chemical Reagent Co., Ltd. (Shanghai, China), which can be used directly. The 0.22 µm filtration membranes were provided by Cinjinghua Co. (Shanghai, China).

### 2.2. Preparation of Reference Extract

Whole fruits of CAVA were picked from the planting bases of Lianyuan Kanglu Biotech Co. Ltd. (Lianyuan, China), which was authenticated as *Citrus aurantium* L. var. *amara* by one of the authors, Yecheng Xiao. Lianyuan has a humid monsoon climate with a hot and humid summer, mild and chilly winter, annual average temperature, sunshine time, and precipitation of 16–17.3 °C, 1538 h, and 1328 mm. The CAVA trees (5-year-old cultivating seedlings in March 2019) were planted by experienced growers in slightly acidic, well-drained sandy loam soil with 4 m between rows and 3.5 m apart within each row. A drip irrigation was performed according to the evapotranspiration. Fertilization was conducted using urea (150 g per tree) in early spring, fermented cattle organic fertilizer 15 kg per tree in the middle of April, 15 kg per tree in early July, and 5 kg per tree in winter. Spirobudiclofen was sprayed in early May. Whole fruits were collected on 10 July 2019, 90 days after flowering, using the uniform random sampling method. Seventy fruits were collected from 10 trees (seven fruits per tree). Collected fruits were sliced immediately, dried at 60 °C in a vacuum oven for 24 h, powdered, and sieved (500 mesh). The powdered sample was stored in a refrigerator at −80 °C. Sample (10.0 g) was extracted by heating reflux with 75% ethanol (*v*/*v*, 80 mL) three times (each for two hours). The filtrated extracts were combined and concentrated at 40 °C using a vacuum rotary evaporation. Finally, 2.3 g of crude extract was obtained.

### 2.3. OLE–DPPH–HPLC−DAD−QTOF-MS Analysis

**Figure 1:**
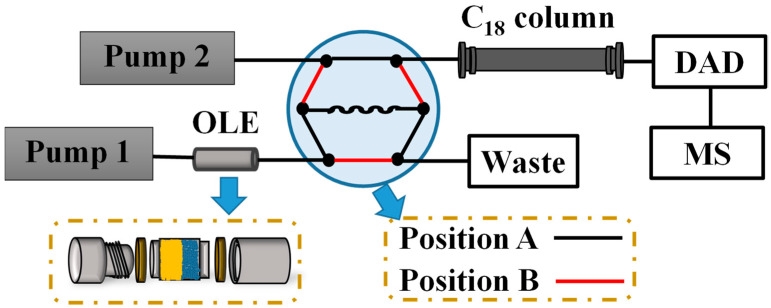
The diagrammatic drawing of OLE–DPPH–HPLC–DAD–QTOF-MS/MS.

The OLE–DPPH–HPLC−DAD−QTOF-MS system was assembled in our laboratory, which included a Shimadzu LC-20AT high pressure constant flow pump (pump 1, Shimadzu Corporation, Kyoto, Japan), a homemade guard column (10 mm length × 4.6 mm i.d.), and an Agilent 1200 liquid chromatography system with a vacuum degasser, quaternary pump (pump 2), manual sampler, DAD (Agilent Technologies, Santa Clara, CA, USA) to assemble (Figure 1). The core part of this technology was the OLE–DPPH system. The guard column was first packed with silica gel (20 mg, 200–300 mesh, Qingdao Haiyang Chemical Co., Ltd., Qingdao, China), followed by ground CAVA (1.0 mg), and then filled with silica gel. After that, the guard column was positioned between pump 1 and the six-way value.

The representative operation program of DPPH–HPLC–DAD–QTOF-MS was performed as follows. First, the six-way value was set at position A. At this time, Agilent ZORBAX SB-C_18_ chromatographic column (250 mm length × 4.6 mm i.d., 5 µm, Agilent, Santa Clara, CA, USA) and Phenomenex C_18_ (4.0 mm length × 3.0 mm i.d., 5 µm, Phenomenex, Torrance, CA, USA) were equilibrated by an initial proportion of mobile phase (methanol: 0.1% formic acid, 25:75, *v*/*v*; 0.8 mL/min) by pump 2, while DPPH solution (0.2 mg/mL, 70% methanol as solvent) was pumped by pump 1 through a guard column with a flow rate at 0.2 mL/min for 2 min. Second, pump 1 was stopped, and extracted compounds containing DPPH were collected by a sample loop (400 µL) and incubated for 10 min. Finally, the six-way value was switched to position B, and compounds were pumped into the C_18_ column for separation and analysis with 0.1% (*v*/*v*) formic acid (A) and methanol (B) as mobile phase in gradient elution mode (0–20 min, 25–55% B; 20–35 min, 55–70% B), column temperature at 25 °C, flow rate at 0.8 mL/min, and detection wavelength at 254 nm. When the DPPH solution was replaced by 70% methanol solution, the crude sample was extracted and analyzed.

The HPLC eluent was split into two streams by an adjustable high-pressure stream splitter (Supelco Port, Bellefonte, PA, USA), and one stream (0.2 mL/min) was introduced into a Bruker compact QTOF-MS (Bruker Co., Bremen, Germany) system with electrospray ionization ion source to obtain structural information. The MS parameters were optimized in positive ion mode: mass range, *m*/*z*, from 100 to 1000; capillary voltage, 3500 V; dry gas (nitrogen) flow rate and temperature, 3.0 L/min and 200 °C; nebulizer pressure, 0.7 bar; end plate offset voltage, 500 V; collision energy, 30 eV for MS/MS analysis.

### 2.4. Antioxidant Activity Evaluation

DPPH scavenging activity was selected to evaluate the antioxidant activity of CAVA crude extract and screened antioxidants, which was performed according to our previous report with minor modifications [21]. Briefly, a series of sample solutions (0.2 mL) were added to DPPH solution (0.04 mg/mL, 1.8 mL), and the mixtures were incubated at 37 °C for 30 min in the dark. Finally, the absorbance of the mixtures was detected at 517 nm. Methanol (0.2 mL) mixing with DPPH solution (0.04 mg/mL, 1.8 mL) served as the control. The percentage of DPPH scavenging ability was calculated as follows: DPPH inhibition rate (%) = (*A*_control_ − *A*_sample_)/*A*_control_ × 100%. The extent of inhibition was recorded as the sample concentration leading to 50% inhibition (IC_50_).

### 2.5. DFT Calculations

Electrons and hydrogen atoms transfer mechanism are always selected to elucidate the radical scavenging procedures [22]. Here, DFT calculations were used to elucidate the electron transfer mechanism. The geometrics and energy levels of ground state for flavonoids were determined using Gauss 9.0 package with B3LYP/6-311+G (d,p) basis sets.

## 3. Results and Discussion

### 3.1. OLE–DPPH–HPLC−DAD−QTOF-MS System Setup

The online system for rapid and efficient screening and identification of antioxidants from CAVA consisted of four elements, i.e., OLE for rapid extraction, online precolumn DPPH reaction for antioxidant screening, HPLC for separation, DAD and QTOF-MS for structural identification. The OLE and online precolumn DPPH reaction were the critical elements of the system.

#### 3.1.1. Optimization of the OLE–HPLC System

**Figure 2:**
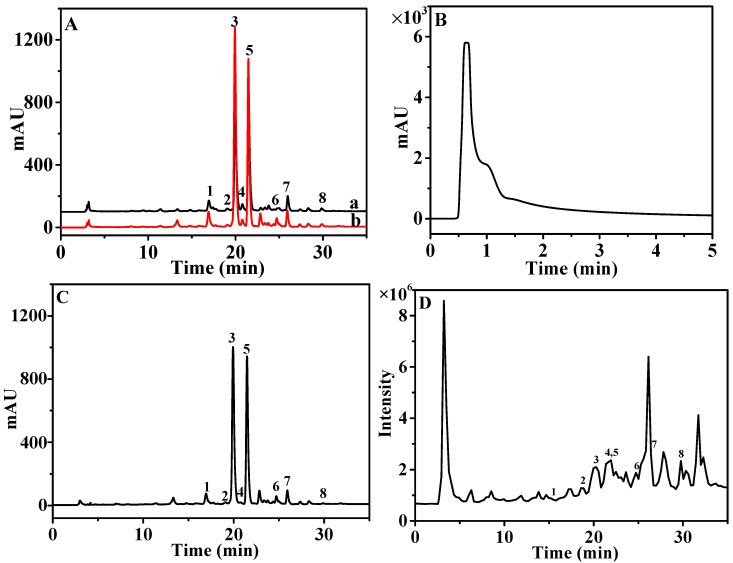
(**A**) HPLC chromatograms at 254 nm for CAVA (20 μL, 11.5 mg/mL) (a) and 1.0 mg of dried CAVA (b); (**B**) chromatogram of CAVA-based OLE; (**C**) OLE–DPPH–HPLC chromatogram at 254 nm for CAVA (1.0 mg); (**D**) total ion current (TIC) chromatogram for CAVA (1.0 mg) in positive ion mode.

The HPLC system was first optimized to achieve a higher resolution for CAVA analysis. At this time, crude extract solution of CAVA (20 μL, 11.5 mg/mL, equal to 1.0 mg of dried CAVA) was selected for the HPLC system. Flavonoids, a type of polyphenol, are the main chemical compounds in the *Citrus* genus; thus, acid is added to the mobile phase to reduce peak tailing and improve resolution [23]. Then, the mobile phase compositions (methanol–water and acetonitrile–water systems with different concentrations of acetic acid or formic acid), elution programs (gradient elution with different initial concentrations), column temperature, flow rate, and detection wavelength were optimized. The results indicated that major compounds in CAVA were well separated and analyzed using 0.1% (*v*/*v*) formic acid (A) and methanol (B) at a flow rate of 0.8 mL/min in a gradient mode (0–20 min, 25–55% B; 20–35 min, 55–70% B), column temperature at 25 °C, and detection wavelength at 254 nm (Figure 2A).

Our previous work found that the OLE system can be used for efficient online extraction [23]. Here, solid CAVA (1.0 mg) was packed in the guard column, and silica gel was selected to fill it. To obtain a high and reproducible recovery, the extraction solvent (i.e., methanol–water with different percentages) and flow rate were investigated and compared. At this time, an additional Shimadzu LC-20AT DAD (Shimadzu Corporation, Kyoto, Japan) was positioned between the guard column and sample loop to record the UV spectrum (254 nm) of OLE. As shown in Figure 2B, compounds in CAVA can be extracted by 70% methanol within 2 min when the flow rate was 0.2 mL/min. Thus, a sample loop (400 µL) was chosen to capture the extracted compounds. A representative OLE–HPLC chromatogram is shown in Figure 2A. Obviously, by comparison with heating reflux extraction, OLE exhibited higher extraction efficiency in terms of the peak areas, perhaps because of the existence of pressure and consecutive extraction behavior on the OLE system. During five parallel OLE–HPLC operations, the RSD value for peak areas was calculated to be 4.61%, indicating high repeatability. Notably, OLE showed overwhelming advantages, that is facile operation, less sample consumption (1.0 mg), less extraction solvent (0.4 mL), shorter extraction time (2.0 min), and higher extraction efficiency. As a result, the OLE system presents irreplaceable superiority in natural product analysis, especially for those precious samples.

#### 3.1.2. OLE–DPPH–HPLC Assay

When DPPH reacted with antioxidants, hydrogen atoms of antioxidants were transferred to DPPH. At this time, the solution color changed from purple (maximum absorbance at approximately 517 nm) to yellow, and the HPLC peak intensity of antioxidants disappeared or reduced. Thus, the DPPH-based scavenging ability assay is always selected to evaluate the antioxidant activity of purified compounds or crude extract [24,25]. Here, the antioxidant activity of CAVA was assessed by DPPH scavenging ability, and the IC_50_ was calculated to be 1.39 ± 0.06 mg/mL. Phenolic compounds are responsible for the antioxidant activity of the *Citrus* genus [26,27,28], and there exists a significant negative correlation between phenolic compounds contents and IC_50_ values [29]. Therefore, CAVA presented a lower IC_50_ value, which showed the presence of antioxidant phenolic compounds with higher contents.

Currently, two kinds of methods based on HPLC and DPPH (i.e., offline precolumn DPPH–HPLC analysis, online postcolumn HPLC–DPPH analysis) have been developed and successfully applied to screen and analyze antioxidants from complex natural products [10,13]. The offline precolumn DPPH–HPLC analysis was shown to be facile, sensitive, and less DPPH consuming [13]. However, tedious sample pretreatment procedures taking at least several hours (e.g., extraction, concentration, dissolution, and incubation) were not avoided. Then, the OLE–DPPH–HPLC system was required. DPPH concentration and incubation time significantly affected the sensitivity. By optimization, the DPPH concentration (0.2 mg/mL) and reaction time (10 min) were determined, and OLE–DPPH–HPLC chromatography is presented in Figure 2C. By comparison with the OLE–HPLC chromatogram in Figure 2A, the peak areas of compounds **1**–**8** are obviously reduced. The more reduced the peak area, the stronger the antioxidant ability of the compound. Thus, antioxidant compounds **1**–**8** were further identified, verified, and quantified.

#### 3.1.3. HPLC–QTOF-MS Analysis

QTOF-MS can generate unambiguous elemental compositions and multiple stages of fragmentation pattern information with high resolution. Therefore, QTOF-MS coupling with HPLC has been widely and successfully applied in natural product research for structural identification and quantification [30,31,32,33].

QTOF-MS parameters were then optimized to achieve higher sensitivity and rich ion signals for structural identification. The screened antioxidants presented quasi-molecular [M + H]^+^ ions and sufficient fragment ions in positive ion mode when the QTOF-MS parameters were set as follows: mass range, *m*/*z*, from 100 to 1000; capillary voltage, 3500 V; dry gas (nitrogen) flow rate and temperature, 3.0 L/min and 200 °C; nebulizer pressure, 0.7 bar; end plate offset voltage, 500 V; collision energy, 30 eV for MS/MS analysis. The total ion chromatogram (TIC) of CAVA is shown in Figure 2D.

### 3.2. Identification of Antioxidants in CAVA

**Figure 3:**
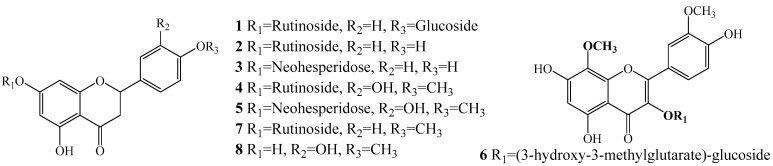
Structures of potential antioxidants in CAVA.

Flavanone glycosides are the main bioactive compounds in the *Citrus* genus, and present one maximum UV absorption peak at approximately 284 nm [15,34]. In QTOF-MS/MS, flavanone glycosides provided higher molecular weight, and their spectra could clearly provide information for the types of aglycone and glycosyl moieties. To date, aglycone fragments reported in positive ion mode in the *Citrus* genus have focused on *m*/*z* at 273.0763 (C_15_H_13_O_5_, naringenin), 287.0919 (C_16_H_15_O_5_, isosakurane), and 303.0869 (C_16_H_15_O_6_, hesperitin) [34,35]. Disaccharide groups are usually linked with C-7 by *O*-glycosidic linkage, and the loss of 308 Da (146 + 162 Da) indicated the presence of *O*-linked rutinoside or neohesperidoside structures [34,35]. Thus, by systematic analysis of their HPLC–DAD–Q–TOF-MS behavior the structures of eight antioxidants were identified. Table 1 lists their retention time, UV, and MS/MS information, while Figure 3 displays their structures.

Compounds **1**–**5**, **7**, and **8** exhibited characteristic UV spectra of flavanone. Compounds **2** and **3** were a pair of isomers with quasi-molecular ions at *m*/*z* 581.1882 ([M + H]^+^, C_27_H_33_O_14_), which showed the same fragment ions at *m*/*z* 435.1257 ([M + H − 146]^+^) and 273.0794 ([M + H – 146 − 162]^+^). Thus, compounds **2** and **3** were unambiguously identified as narirutin and naringin by comparison of their retention time and MS/MS spectra of standards. Similarly, isomeric **4** and **5** were unequivocally confirmed as hesperidin and neophesperidin. Compound **1** exhibited a parent ion at *m*/*z* 743.2375 ([M + H]^+^, C_33_H_43_O_19_, 162 Da greater than that of **2**), indicating an additional glucosyl group for **1**. In the MS/MS spectra, fragment ions at *m*/*z* 581.1853 ([M + H − 162]^+^, the same as the parent ion for **2**), 435.1280 ([M + H – 162 − 146]^+^), and 273.0747 ([M + H – 162 – 146 − 162]^+^) might come from previously reported naritutin-4′-*O*-glucoside [15]. Compound **7** showed an [M + H]^+^ ion at *m*/*z* 595.1933 (C_28_H_35_O_14_), and fragment ions at *m*/*z* 449.1433 ([M + H − 146]^+^) and 287.0911 ([M + H – 146 − 162]^+^), all of which were 14 Da greater than that of **2**. It can be reasonably concluded that one hydroxyl group in **2** has been replaced by a methoxyl group to form **7**. Thus, by coeluting with a standard, compound **7** was easily assigned as didymin. Compound **8** with less polarity exhibited a quasi-molecular ion at 303.0854 ([M + H]^+^, C_16_H_15_O_6_) and a [^0,2^B]^+^ ion at *m*/*z* 153.0174, and its HPLC–DAD–QTOF-MS behavior was consistent with the known standard, hesperitin.

Compound **6** showed two maximum UV absorption wavelengths at 276 and 340 nm, which were the characteristic spectra for flavonols. Compound **6** gave a [M + H]^+^ parent ion at *m*/*z* 653.1686 (C_29_H_33_O_7_). The specific fragment ions at *m*/*z* 509.1389 ([M + H − 144]^+^) and 347.0749 ([M + H – 144 − 162]^+^), indicated the existence of a (3-hydroxy-3-methylglutarate) glucosyl group. Thus, compound **6** was tentatively assigned as Limocitrin-3-O-(3-hydroxy-3-methylglutarate)-glucoside by comparison with that reported in *C. limon* and *C. Paradisi* cv. Changshanhuyou [15].

### 3.3. Quantification of Antioxidants in CAVA

As is well known, natural compounds always achieve their beneficial effects by holistic interactions of numerous effective major and minor compounds, and the contents of bioactive compounds are closely related to their beneficial effects. Here, six commercially obtained flavanones (**2**–**5**, **7**, and **8**) were selected for quantitative analysis using HPLC–QTOF-MS for the first time. The method was validated by linearity, the limit of detection (LOD, signal-to-noise ratio, *S*/*N* = 3), matrix effect, precision, and recovery (Table 2). All six flavanones presented good linearity in the detected concentrations with *R*^2^ greater than 0.994, while the LOD values ranged from 0.09 to 0.68 μg/mL. Matrix effects ranging from 94.0% to 105.4% were determined, indicating that no noticeable ion enhancement or ion suppression existed. The precision was estimated by five consecutive injections of mixed standard samples each day within five consecutive days. The intraday relative standard deviations (RSD) were between 2.7% and 4.6%, and those for interday RSD were between 4.5% and 8.6%, which indicated the high precision. The accuracy was evaluated by mixing 2.0 mg/g of mixed standards into the CAVA sample, and acceptable recoveries were obtained in the range of 95.1–105.2%. The results indicated that the developed HPLC–QTOF-MS method was sensitive, precise, and accurate enough for the quantification of six flavanones in CAVA.

Subsequently, the contents of six flavanones in CAVA were evaluated (Table 2). Naringin (50.37 ± 0.43 mg/g) and neohesperidin (38.20 ± 0.27 mg/g) were the predominant compounds in CAVA, which were significantly higher than narirutin (0.62 ± 0.07 mg/g), hesperidin (1.49 ± 0.04 mg/g), didymin (3.91 ± 0.03 mg/g), and hesperitin (0.73 ± 0.06 mg/g). Specifically, naringin and neohesperidin are the official markers to monitor the quality of CAVA [16].

### 3.4. Antioxidant Activity Evaluation

The DPPH scavenging activities of compounds **2**–**5**, **7**, and **8** were evaluated (Table 3). The aglycone hesperitin (**8**) expressed stronger antioxidant activity with IC_50_ at 39.07 ± 2.51 μg/mL. Phenolic hydroxyl groups are the source of antioxidant activities; thus, *O*-glycosylation can decrease their antioxidant capacities [36]. Here, flavanone glycosides (**2**–**5** and **7**) presented potent antioxidant activities with IC_50_ vales from 111.9 ± 10.06 to 361.50 ± 13.29 μg/mL. Flavanone glycosides have been reported widely and abundantly in the *Citrus* genus [37]; therefore, the *Citrus* genus can be an alternative source of organic antioxidants. Furthermore, the *Citrus* genus is famous worldwide, and oral consumption of them at commonly used doses had a favorable safety and tolerability profile [38]. Thus, the increased consumption of the *Citrus* genus may provide health benefits.

### 3.5. DFT Calculations

**Figure 4:**
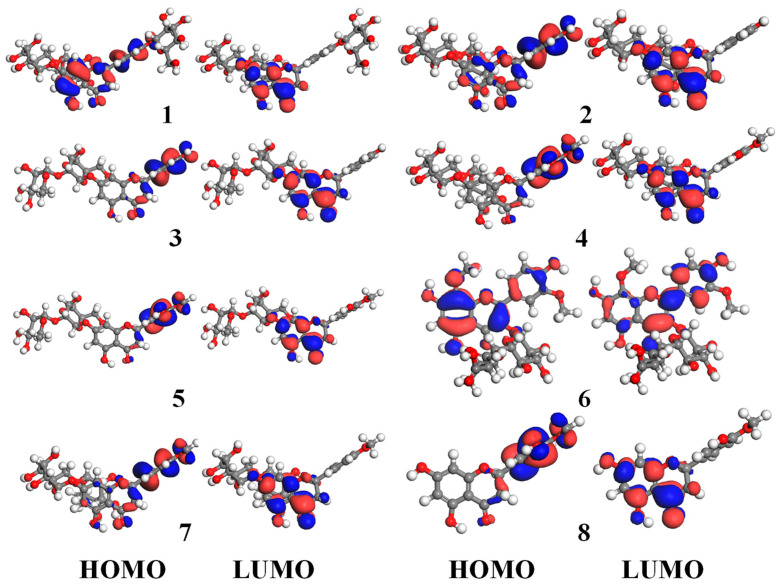
TD-DFT calculations of screened flavonoids.

The Frontier Molecular Orbital theory is always selected to quantitatively predict the electron transport ability and chemical reactivity of molecules. The Highest Occupied Molecular Orbital (HOMO) and the Lowest Unoccupied Molecular Orbital (LUMO) are two key molecular orbitals for elucidation of chemical reactivity and the action. The HOMO contains the valence electrons and presents the highest energy, which can donate the electrons easily. On the contrary, the LUMO has the electron-deficient capability, which tends to accept electrons. The interaction between HOMO and LUMO was the main factor to form a transition state. Thus, HOMO and LUMO energies, and HOMO–LOMO energy gap (E_g_) can provide important information for elucidation of chemical reactivity of compounds. It was reported that molecules with higher HOMO energy levels and lower E_g_ will transfer electrons easily and exhibited higher antioxidant activities [22]. Here, DFT calculations by Gauss 9.0 package were investigated, and Table 3 shows the calculated HOMO, LOMO, and E_g_ of eight flavonoids, while Figure 4 presents the HOMO and LUMO of them. As showcased in Figure 4, the electron cloud is essentially concentrated on the B-ring of flavonoids on the HOMO level, which indicates that the B-ring tends to donate electrons. However, the electron cloud focuses on the A and C-rings on the LUMO level, which shows that this region is susceptible to accepting electrons. Among flavanones (**1**–**5**, **7**, and **8**), aglycone **8** exhibited the higher highest HOMO energy (−6.123 eV) and the lowest E_g_ (4.292 eV) compared to glycosides (**1**–**5** and **7**) with HOMO energies from −6.545 to −6.128 eV and Eg ranges from 4.441 to 4.779 eV, respectively (Table 3). The results implied that flavanone aglycone might contain better electron transport ability and better antioxidant activity than flavanone glycoside. As observed for **2**–**5**, and **7**, variations in HOMO energies and Eg presented good correlations with their antioxidant activities.

## 4. Conclusions

In this study, a new integrated system, OLE–DPPH–HPLC−DAD−QTOF-MS, was fabricated for facile, rapid, and efficient screening and identification of antioxidants from complex natural products. Within this system, sample extraction, antioxidant evaluation, chromatographic separation, and structural identification can be successfully performed in a single run within 45 min. The time-, labor-, energy-, and reagent-consuming procedures for sample pretreatment and bioactive compounds screening were avoided. As a result, eight antioxidants were screened and identified in CAVA. A precise and reliable quantitative method was developed for the successful analysis of six antioxidant flavanones in CAVA. DFT studies presented the electronic variables, HOMO and LUMO energies, and E_g_ for evaluation of chemical reactivity of screened flavonoids. The developed online system might simplify and accelerate antioxidant screening in complex natural products. Furthermore, elucidation and evaluation of antioxidants in CAVA provides a theoretical foundation and scientific basis for its quality control, and clinical and daily utilization.

## Figures and Tables

**Table 1:** Identified antioxidants in CAVA and their DPPH scavenging activity evaluation (IC_50_, μg/mL).

No.	*t*_R_ (min)	λ_max_ (nm)	[M + H]^+^ (∆ ppm)	Formula	Fragment Ions (*m*/*z*)	Identification
**1**	16.9	287	743.2375 (−3.0)	C_33_H_42_O_19_	581.1853 [M + H − Glu]^+^ 435.1280 [M + H − Rut]^+^ 273.0747 [M + H – Glu − Rut]^+^	Narirutin-4′-*O*-glucoside
**2**	19.1	284	581.1882 (2.1)	C_27_H_32_O_14_	435.1257 [M + H − Rha]^+^ 273.0794 [M + H − Rut]^+^	Narirutin
**3**	19.9	284	581.1852 (−3.1)	C_27_H_32_O_14_	435.1284 [M + H − Rha]^+^ 273.0749 [M + H − Neo]^+^	Naringin
**4**	20.8	286	611.1984 (1.3)	C_28_H_34_O_15_	465.1412 [M + H − Rha]^+^ 303.0886 [M + H − Rut]^+^	Hesperidin
**5**	21.4	284	611.1948 (−4.6)	C_28_H_34_O_15_	465.1373 [M + H − Rha]^+^ 303.0850 [M + H − Neo]^+^	Neohesperidin
**6**	24.7	276 340	653.1686 (−4.9)	C_29_H_32_O_17_	509.1389 [M + H − 144]^+^ 347.0749 [M + H – 144 − Glu]^+^	Limocitrin-3-O-(3-hydroxy-3- methylglutarate)-glucoside
**7**	26.0	284	595.1993 (−5.7)	C_28_H_34_O_14_	449.1433 [M + H − Rha]^+^ 287.0911 [M + H − Rut]^+^	Didymin
**8**	29.9	286	303.0854 (−4.9)	C_16_H_14_O_6_	153.0174 [^0,2^B]^+^	Hesperitin

**Table 2:** Calibration curves, linearity, LOD, LOQ, matrix effect, precision, recovery, and contents for six antioxidants in CAVA.

Compd	Regression Equation ^a^	*R* ^2^	Linear Range (μg/mL)	LOD (μg/mL)	Matrix Effect (%)	Precision (n = 5) (RSD, %)		Recovery ^b^ (%)	Contents (mg/g) ^c^
						Intraday	Interday		
**2**	*y* = 389.92*x* + 563.80	0.997	1.0–100	0.17	98.2	3.1	8.6	95.1	0.62 ± 0.07
**3**	*y* = 349.11*x* + 170.89	0.995	3.0–400	0.68	100.3	4.5	5.9	99.2	50.37 ± 0.43
**4**	*y* = 510.94*x* + 86.52	0.999	1.0–100	0.25	104.7	3.5	4.5	101.9	1.49 ± 0.04
**5**	*y* = 635.27*x* + 91.49	0.994	3.0–400	0.50	96.3	4.6	6.3	105.2	38.20 ± 0.27
**7**	*y* = 434.95*x* + 131.84	0.995	3.0–300	0.59	94.0	3.2	5.7	96.4	3.91 ± 0.03
**8**	*y* = 873.60*x* − 49.25	0.999	0.5–20	0.09	105.4	2.7	4.8	94.7	0.73 ± 0.06

^a^*y* = A*x* + B, *y* is the peak area; *x* is the concentration of the detected compounds (µg/mL). ^b^ Addition concentration for all compounds at 2.0 mg/g. ^c^ Data are represented as the mean value ± SD, n = 3.

**Table 3:** DPPH scavenging activity, HOMO energy (E_HOMO_), LUMO energy (E_LUMO_) and HOMO-LUMO gap (E_g_) in eV of eight screened flavonoids.

Compounds	IC_50_	E_HOMO_	E_LUMO_	E_g_
**1**	- ^a^	−6.545	−1.766	4.779
**2**	257.06 ± 9.32	−6.470	−1.761	4.709
**3**	111.9 ± 10.06	−6.128	−1.687	4.441
**4**	361.50 ± 13.29	−6.482	−1.767	4.715
**5**	178.55 ± 11.28	−6.139	−1.565	4.574
**6**	- ^a^	−5.924	−2.029	3.895
**7**	219.73 ± 16.45	−6.438	−1.765	4.673
**8**	39.07 ± 2.51	−6.123	−1.831	4.292

^a^ Not detected.

## Data Availability

Data is contained within the article.
